# Criteria for selecting microhaplotypes: mixture detection and deconvolution

**DOI:** 10.1186/s13323-014-0018-3

**Published:** 2015-01-28

**Authors:** Kenneth K Kidd, William C Speed

**Affiliations:** Department of Genetics, Yale University School of Medicine, 333 Cedar Street, PO Box 208005, New Haven, CT 06520-8005 USA

**Keywords:** Microhaplotype, DNA mixtures, Forensic identification, Population genetics

## Abstract

**Background:**

DNA sequencing is likely to become a standard typing method in forensics in the near future. We define a microhaplotype to be a locus with two or more single nucleotide polymorphisms (SNPs) that occur within a short segment of DNA (e.g., 200 bp) that can be covered by a single sequence run and collectively define a multiallelic locus. Microhaplotypes can be highly informative for many forensic questions, including detection of mixtures of two or more sources in a DNA sample, a common problem in forensic practice.

**Results:**

When all alleles are equally frequent, the probability of detecting three or more alleles in a mixture is at maximum. The classical population genetics concept of effective number of alleles at a locus, termed A_e_, converts the unequal allele frequencies at a locus into a value that is equivalent to some number of equally frequent alleles, allowing microhaplotype loci to be ranked. The expectations for the ability to qualitatively detect mixtures are given for different integer values of A_e_, and the cumulative probabilities of detecting mixtures based on testing multiple microhaps are shown to exceed 95% with as few as five loci with average A_e_ values of even slightly greater than 3.0.

**Conclusions:**

Microhaplotypes with A_e_ values of >3 will be exceedingly useful in ordinary forensic practice. Based on our studies, 3-SNP microhaplotypes will sometimes meet this criterion, but 4-SNP microhaplotypes can even exceed this criterion and have values >4.

**Electronic supplementary material:**

The online version of this article (doi:10.1186/s13323-014-0018-3) contains supplementary material, which is available to authorized users.

## Background

With the arrival of inexpensive DNA sequencing appropriate for forensic applications, we have reexamined the optimal type of genetic marker for forensic applications. It is clear that several relevant forensic questions cannot be answered by the existing or expanded set of short tandem repeat polymorphisms (STRPs) used by combined DNA index system (CODIS) [1,2]. The CODIS loci or their equivalent STRPs in other countries are good for individual identification, their original and still primary use. While most CODIS loci can be adequately genotyped by sequencing, massively parallel sequencing (MPS) methodology allows other types of markers to be included in the genotyping.

Single nucleotide polymorphisms (SNPs) can enhance the individual identification statistics beyond what the STRPs provide either as nucleotide variants within individual STRP alleles or as a separate set of markers [3-5]. By their multiallelic nature STRPs both provide familial relationship information and also can be used to identify and resolve mixtures of DNA from two or more individuals in a single sample. Unfortunately, individual di-allelic SNPs can provide only weak evidence of familial relationships or resolution of mixtures precisely because there are only two alleles. However, sets of SNPs and/or small insertion-deletion polymorphisms (DIPs or Indels) can provide robust information on biogeographic ancestry [6-10], a type of investigative information that panels of STRPs, such as the CODIS loci, cannot provide due to their high global heterozygosity and greater mutation rate. Some SNP genotypes are highly correlated with physical phenotypic traits such as pigmentation of skin/hair/eye [11-13], another form of information that STRPs cannot provide.

Our lab has long used minihaplotypes (minihaps) in anthropological studies [14-16]. These genomic regions of 10 kb or longer with multiple SNPs of high heterozygosity and minimal intermarker recombination can have great value for ancestry determination and anthropology, since the statistical inference of phase can be very accurate. The two areas of forensic analysis that individual SNPs and minihaps cannot address well, familial/lineage information and mixture detection and deconvolution, can be addressed by a new type of marker, SNPs grouped into microhaplotypes (microhaps). Microhaps are defined as loci of two or more SNPs within the span of a single sequence run (arbitrarily set currently at 200 bp) with three or more common allelic combinations (haplotypes) of the SNPs [17,18]. Microhaps properly selected to also avoid recombination hot spots will have mutation rates much lower than those of the STRPs.

In our initial studies [17,18] of many multiallelic microhaps, we have shown that when genotyped by MPS, microhaps can fulfill all the forensic objectives for which the STRPs were originally selected and are now used. Use of MPS obviates the problems of multiplex-ability, sensitivity, and assay cost associated with individual SNP genotyping. In addition to being useful for identification and lineage/family relationships, microhaps can provide information on biogeographic ancestry and can be useful for both detecting and deconvoluting mixtures of DNA. The issue now is identifying and fully characterizing a set of microhaps with the optimum characteristics for specific purposes. We are screening existing databases for appropriate candidate regions and then testing our own panel of 54 population samples to confirm and expand the data. Only microhaps with three or more alleles are of value, and many close SNPs have complete linkage disequilibrium (LD) with only two extant haplotypes (alleles) (cf. discussion in [18]). Given the potentially high heterozygosity of microhaps with three or more alleles, the genotypic uniqueness of individuals for identification is not a major issue: random match probabilities can easily be below 10^−30^ with fewer than 50 loci [18]. However, the optimal characteristics of loci differ for the other purposes, as we have explained in developing our panels of single SNPs for identification [4,19] and ancestry [5]. For ancestry inference, the allele frequencies of the loci used must show variation among the populations being considered. For lineage studies, the loci should be highly heterozygous with multiple alleles; yet, that criterion alone may not be sufficient for optimal detection of mixtures.

## Methods

### Developing criteria for selecting loci to detect mixtures

Heterozygosity is the maximum possible when all alleles are equally frequent, and this clearly is optimal for individual identification for any specified number of alleles. For ancestry inference, high heterozygosity is less important than frequency variation among populations. However, in using large datasets generated from either chip-based genotyping or from whole-genome MPS, we have found that many of the loci that have the highest variation among populations represent either genotyping error or assembly errors in MPS analysis for the population(s) with more deviant frequencies. For lineage and ancestry inference, identity by descent (IBD) is important. Loci that may be hypervariable due to frequent mutation and/or recombination would complicate determining IBD among extended relatives or within a tribe of moderate size. Therefore, we have chosen to focus on loci that have most alleles at >5% (arbitrarily chosen); a frequency greater than frequent recombination is likely to generate and a frequency greater than typing errors. We could also limit selection to loci that have only the *n +* 1 alleles that can be generated by *n* accumulated mutations. However, we often see an allele (haplotype) that could only have arisen by a crossover among the SNPs within a microhap. In many cases, those appear to be single historical crossover products that have drifted to high frequency and are not recurring at a meaningful frequency of, say, >10^−4^. These considerations help us avoid pursuit of loci that may be hypervariable due to frequent mutation, frequent recombination, and/or errors in the datasets screened.

**Figure 1:**
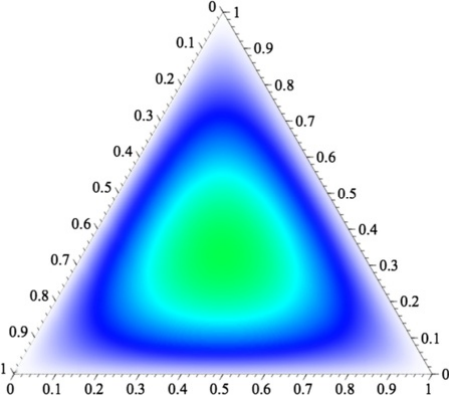
**Ternary plot of the probability of a qualitatively detectable mixture.** The probability of having more than two haplotypes present, for a three locus system with allele frequencies of p, q, and r, is calculated for a set of genotypes from a random pair of individuals. The values range from zero along the margins with only two alleles present to the maximum at the “center” where all alleles are equally frequent.

Given the above consideration, how do we maximize the ability to detect mixtures? We are interested in qualitative determination that a mixture is present in a forensic sample. That occurs when three or more distinct alleles are observed. Obviously, an infinite number of alleles maximizes uniqueness of individuals and hence the difference between any two individuals. To consider the issue for a small finite number of alleles, we have started with the simplest case of three alleles. To evaluate the probability of a mixture having more than two alleles present, we have simply used a multinomial expansion to calculate the probability of a mixture of DNA from two independent individuals having at least three different alleles as a function of the allele frequencies. The function for three alleles is simply (*p* + *q* + *r*)^4^ = (*p* + *q* + *r*)^2^ (*p* + *q* + *r*)^2^, which is the product of the Hardy-Weinberg genotype arrays for two random unrelated individuals. As shown in Figure 1, the sum of the terms in which three alleles occur is maximized when the alleles are equally frequent. This is also the situation that maximizes heterozygosity. When there are four alleles, (*p* + *q* + *r* + *s*)^4^, the maximum occurs when all alleles are equally frequent. Conceptually, this maximum occurs at the center of a tetrahedron with each face identical to Figure 1. The overall pattern generalizes to any number of alleles. Table 1 gives those maximum values for loci with 3, 4, and 5 equally frequent alleles. The maximum probability of three or more alleles in a mixture occurs when all alleles at a locus are equally frequent. Obviously, with multiple loci, the maximum probability will increase as a function of the values at each individual locus.

**Table 1:** **Maximum probabilities of detecting a mixture of two random unrelated individuals for an N-allele microhap**

**Number of equally frequent alleles**	**Probability of three alleles being present**	**Probability of four alleles being present**	**Total probability of detecting more than two alleles in a mixture**
Three	0.4444	-	0.4444
Four	0.5625	0.09375	0.65625
Five	0.5760	0.1920	0.7680

Maximum probabilities of detecting a mixture of two random unrelated individuals for three-, four-, and five-allele microhaps. These are the values when all alleles are equally frequent. As shown in Figure 1, the values are lower when the frequencies are not equal.

In searching the HGDP [20,21] or 1000 Genomes [22] databases for optimal microhaps, we have often found either relatively uninformative loci with only one or two really common alleles or many alleles in very unequal frequencies. The loci with only two alleles are easily discarded but the question remains of how to rank the loci for mixture detection when there are multiple alleles at very different frequencies. Classical population genetics gives the answer with the concept of effective number of alleles [23] which we symbolize as A_e_. A_e_ is defined for a locus as the equivalent number of equally frequent neutral alleles in terms of population dynamics. It is calculated as the reciprocal of the homozygosity: $$ \raisebox{1ex}{$1$}\!\left/ \!\raisebox{-1ex}{${\displaystyle \sum {p}_i^2}$}\right. $$ where *p*_*i*_ equals the frequency of allele *i* and summation is over all alleles at the locus.

We know that the potential to detect a mixture increases with more equally frequent alleles (Table 1). Using effective number of alleles converts each locus to the same “standard” and the higher the effective number of alleles, the more probable a mixture could be detected. This applies to a single population but one must also consider variation among populations since the forensic caseload potentially involves individuals from populations originating from many different parts of the world. Therefore, we are using our large set of populations to confirm the variation seen in the initial screenings as well as to extend knowledge to many additional populations.

The A_e_ calculation allows the ranking of loci by their probabilities of qualitatively detecting a mixture. In reality, several loci will be used in a forensic analysis. If we select loci with an average A_e_ of 4, then we can calculate the probability of “detecting” a mixture with at least one of *n* loci as 1-(1–.65625)^*n*^. Table 2 gives the probability of “detecting” a mixture qualitatively for different numbers of loci studied at integral values of A_e_ from 3 to 5. Obviously, mixtures of loci with different A_e_ values will give intermediate results as will loci with non-integer A_e_ values between 3 and 5. We have not extended the table beyond 5 loci since the probability rapidly approaches certainty and we have yet to find a locus with a global average A_e_ of 5 or greater. Nor have we considered numerically the issue of a mixture of more than two individuals, but the logic applies to such cases as well and detecting five alleles indicates at least a three person mixture.

**Table 2:** **Cumulative probability of a mixture having three or more alleles at two or more loci**

	**Number of loci studied**			
**Effective number of alleles, A**_**e**_	**2**	**3**	**4**	**5**
3	0.69131	0.82849	0.90471	0.94706
4	0.88184	0.95938	0.98604	0.9952
5	0.94618	0.98751	0.99710	0.99933

Cumulative probability of a mixture having three or more alleles at two or more loci, for integral values of A_e_. See text.

## Results and discussion

**Figure 2:**
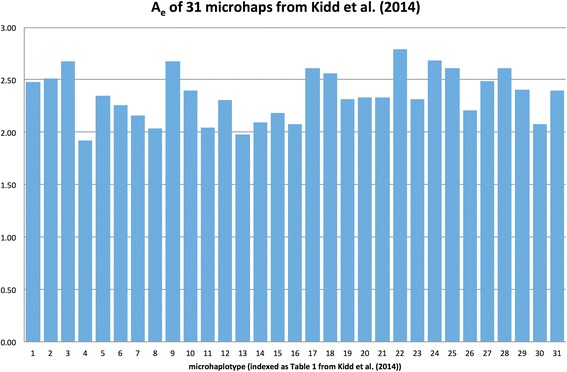
**Histogram of A**_e_ **for the original 31 microhaps published in** **[** 18 **]** **.**

After identifying a potentially useful microhap in large public data sets, we used our set of DNA samples from over 2,500 individuals originating from 54 populations to confirm the pattern of variation and obtain statistics from a global sample of populations [5]. Our initial studies involved microhaps with only two or three SNPs [17,18]. We started with those because they were the simplest to find, often involving SNPs we had already tested on the 54 populations we are routinely studying. The global average A_e_ for the 31 microhaps published [18] ranges from 1.9 to 2.8 (Figure 2). When we recognized that the maximum power to detect mixtures is a function of A_e_, we decided to use A_e_ as an effective way to compare loci and to focus on microhaps with an A_e_ >3.0. Microhaps with four SNPs are an interesting subset to consider because they can have an A_e_ considerably greater than 3.0. While there are potential microhaps with more than four SNPs, we found that the pool of potential microhaps with SNP frequency data for all SNPs in the microhap, and with individual SNP heterozygosities above 18%, was very small. By focusing on 4-SNP microhaps, there still remained a large enough pool to be able to look at frequency distributions and the pairwise correlations between SNP frequencies in the microhaplotype. We developed empiric thresholds based on these distributions. In our search of the 1000 Genomes data using criteria described in Additional file 1, we identified 341 microhaps comprised of four SNPs. We calculated A_e_ for each locus in each population using the haplotype frequencies calculated by PHASE [24]. Considering the matrix of 341 microhaps × 20 populations, the row and column averages show markedly different ranges. The average A_e_ for each of the 341 microhaps ranged from 1.79 to 6.96. The average A_e_ for each of the 20 populations ranged from 2.92 to 3.64.

We are in the process of testing many of these and other microhaps and present here examples of the highest global average A_e_ values among the 2-SNP, the 3-SNP, and the 4-SNP microhaps that we have now tested on over 60 populations (Table 3). The examples range from an A_e_ of 2.7 for the second highest 2-SNP microhap to just over 4.7 for the highest 4-SNP microhap.

**Table 3:** **Examples of 2-SNP, 3-SNP, and 4-SNP microhaplotypes with largest A**_e_ **values**

**Provisional locus name**	**SNPs involved**	**Extent in bp**	**Average global A**_**e**_
Microhap048 (mh24:C14ORF43 [18])	rs12717560	159	2.708
	rs12878166		
Microhap046 (mh22:SUDS3 [18])	rs1503767	72	2.842
	rs11068953		
Microhap049	rs9937467	59	2.888
	rs17670098		
	rs17670111		
MicroHap061	rs763040	146	3.192
	rs5764924		
	rs763041		
MicroTetrad180	rs12802112	193	4.008
	rs28631755		
	rs7112918		
	rs4752777		
MicroTetrad315	rs8126597	145	4.763
	rs6517970		
	rs8131148		
	rs6517971		

Examples of 2-SNP, 3-SNP, and 4-SNP microhaplotypes with largest A_e_ values characterized on our laboratory’s populations to date. The 2-SNP microhaps were published in [18] under the locus name appended to the “Provisional Locus Name” field; The microhap number indicates the number of that locus in [18] and in Figure 2.

**Figure 3:**
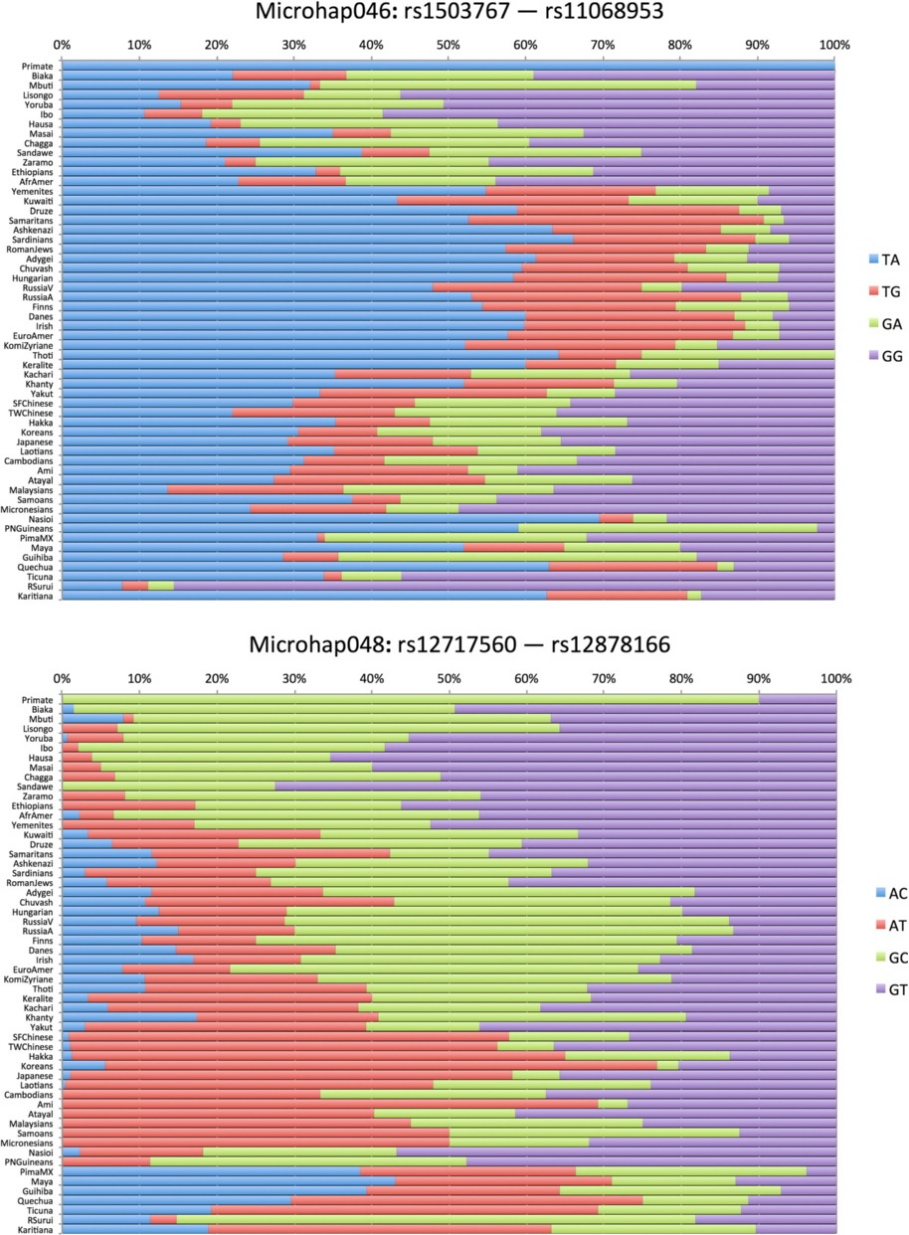
**Haplotype frequency plots for best 2-SNP microhaps characterized to date.**

**Figure 4:**
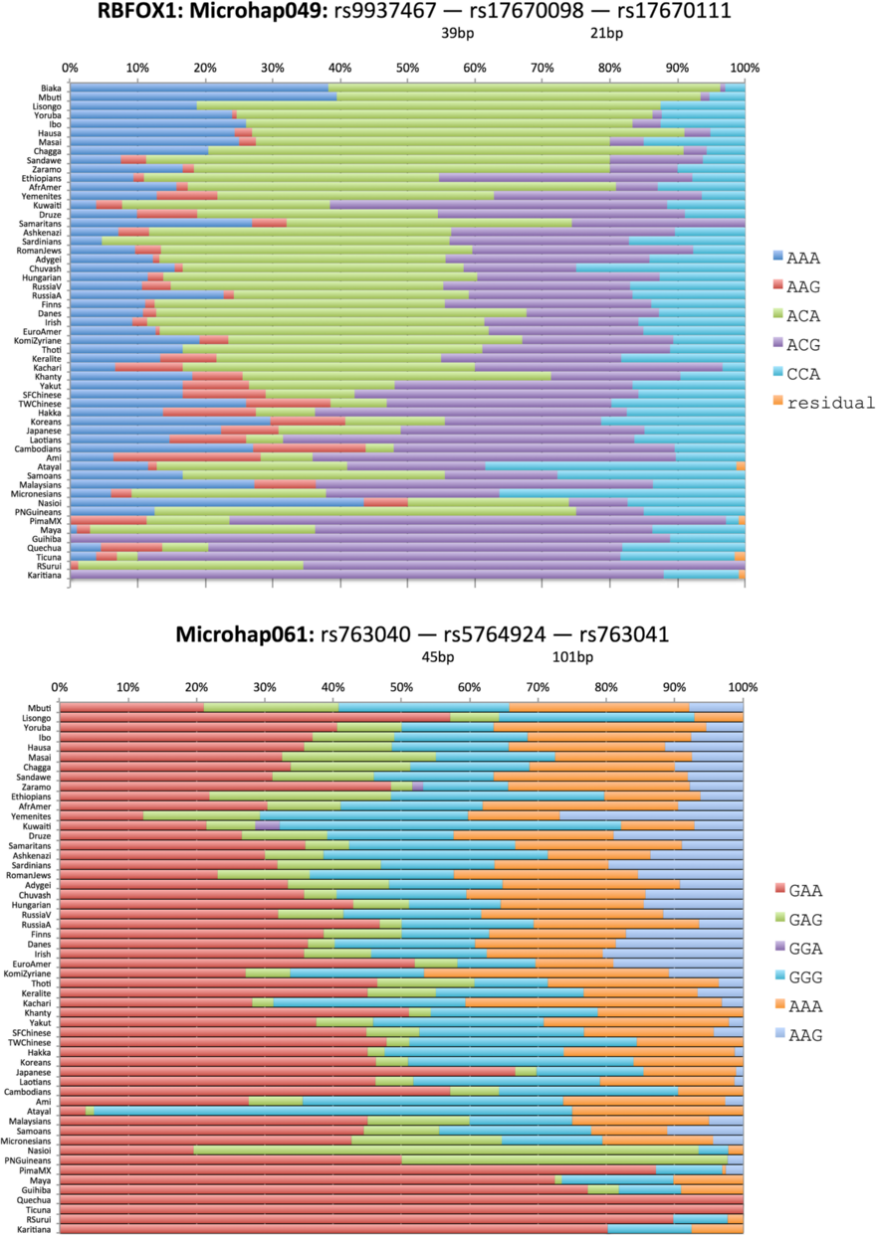
**Haplotype frequency plots for best 3-SNP microhaps characterized to date.**

**Figure 5:**
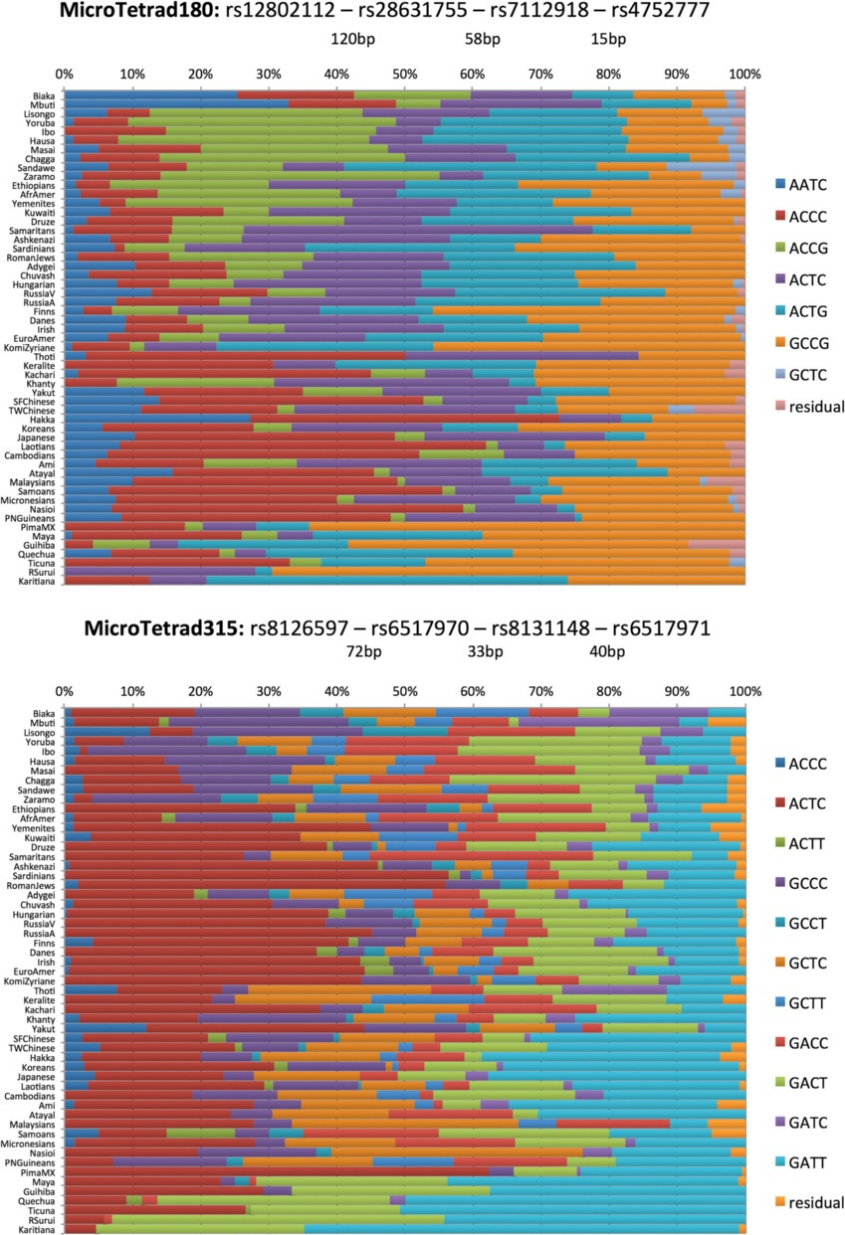
**Haplotype frequency plots for best 4-SNP microhaps characterized to date.**

Figures 3, 4, and 5 show population-specific allele frequencies for the six microhaps in Table 3 for a global set of populations. The allele frequencies clearly vary among populations, some of which reflects sampling error, but clear differences also exist among biogeographic regions for some of these loci. We can rank the loci by the A_e_ for a specific population of interest or, since *a priori* the relevant population(s) may be unknown for a forensic analysis, by the global average A_e._ However, it is important to recognize that by selecting for high average A_e_ to maximize mixture detection, we are tending to reduce large regional differences in allele frequencies and thus choose less ancestry informative loci. In future papers, we will discuss criteria for identifying and evaluating optimal microhaps for ancestry and lineage inference.

With two SNPs and a maximum of four haplotypes, we have yet to find a microhap with a global average A_e_ of >3.0. While 17 of the 27 two-SNP microhaps we have already published [18] have individual populations with A_e_ >3.0, global averages are generally decreased especially due to Native American populations, where decreased genetic diversity means only 4 of the 2-SNP microhaps have A_e_ >3.0 in these populations. Microhap048 has all four possible haplotypes but the unequal frequencies result in a global average A_e_ of only 2.71, the second best of those we have examined. Microhap046 also has all four possible haplotypes and the highest A_e_ for the 2-SNP microhaps; the allele frequencies are seemingly more uneven but the global average A_e_ is slightly larger, 2.84. The 3-SNP microhaps allow for a maximum of eight microhaps, but our selection criteria have generally identified loci with fewer than eight alleles. Microhap049 is the second best with five of the eight possible haplotypes at common frequencies in all regions of the world with an additional microhap seen only in the Americas. The global average A_e_ of 2.89 is barely larger than the best of the 2-SNP microhaps. Microhap061 also has five haplotypes with similar frequencies among most populations except Native Americans and has a global average A_e_ of 3.12. The 4-SNP microhaps we have studied so far allow a much greater number of alleles and the two illustrated show considerable within-population variation. Microhap Tetrad180 has six haplotypes that occur essentially globally, and its A_e_ of 4.008 is the second best. Finally, microhap Tetrad315 has the most allelic complexity with 10 of the 16 possible haplotypes reaching frequencies >5% in many populations and a global average A_e_ of 4.76, the highest global average we have found to date.

Microhap Tetrad315 illustrates our concern about microhaps with frequent recombination for ancestry inference. We have not yet been able to document that most of the variation is old and not frequently regenerated although it is clear that certain combinations among the more common haplotypes are not seen at the frequencies that might occur if recombination were very frequent. Even a high recombination rate of 1% would still allow meaningful lineage inference with several loci such as this. However, that concern is not relevant to individual identification and mixture detection and deconvolution. Those rely on the allele/haplotype frequencies in the population, not on the ongoing origins of the alleles or identity by recent descent of the different alleles. This locus and the other 4-SNP microhap illustrated, Tetrad180, will be able to provide excellent mixture detection ability.

The calculations we have made assume that the contributions from the two individuals can be detected. Issues of sensitivity enter when the contributions are not equal, as is almost always the case in forensics. Those issues are dependent on the method used and, since we assume sequencing will be used, on the depth of coverage, i.e., the number of reads per locus. With an average of >100 reads per allele, a minimal expectation for MPS, a third allele can be said to be present if several reads detect it. With experience, not yet available, the community can decide on the criteria necessary to overcome the stochastic aspect of number of reads of an allele. With a clear stochastic threshold, quantitative estimation of the relative contributions of individuals to a mixture is clearly possible. We note that these concerns are no different from those currently used for the CODIS STRPs. Considerable experience exists with these STRPs yet there are different criteria used in different labs to evaluate whether a low RFU peak represents chance or a real peak. With MPS, even very low levels of a second contributor to a mixture could result in multiple reads with a unique combination of SNP alleles (a unique haplotype) on single reads, giving multiple confirmation of the presence of more than one DNA. In contrast to many evaluations of mixtures using STRPs that require decisions surrounding stutter peaks and low RFU peaks in mixtures, MPS microhaps are clearly superior. We think that sequence will have less ambiguity at specific nucleotides and make low levels of mixture detectable, but actual experience is needed.

To date, we have evaluated candidate microhaps using TaqMan assays to detect the individual SNPs that we know from our database screens will very likely yield useful loci. If we had sequencing data instead of genotype data, we would be able to show greater A_e_ measures due to cryptic variation unseen by our pilot SNP-typing method. A rare SNP documented at 1% most likely subdivides some haplotype. Similarly, even the one-off variants already documented will provide what is likely a rare unique haplotype. From the available data, one cannot estimate how the combinations of these untested SNPs/variants with the tested SNPs will resolve into haplotypes but additional information will exist. For example, Microhap048, a 2-SNP microhap, has additional documented SNPs/variants within that 200 bp. Similarly, MicroTetrad315 has additional variants. The additional variation already documented for these two microhaps is summarized in Table 4.

**Table 4:** **Additional documented variation in Microhap048 and MicroTetrad315**

**Provisional locus name**	**rs number (build 138)**	**Role in microhap**	**Chr.**	**Position GRCh37/hg19**	**Clustered allele frequency**
Microhap048					
	rs149195448		14	74250553	0.006
	rs12717560	SNP 1	14	74250557	0.331
	rs76446474		14	74250562	0.005
	rs374425620		14	74250591	n/a
	rs191001036		14	74250647	0.001
	rs113480934		14	74250694	n/a
	rs12878166	SNP 2	14	74250715	0.377
	rs12879393		14	74250730	0.286
MicroTetrad315					
	rs8126597	SNP 1	21	21880086	0.298
	rs192464415		21	21880096	0.001
	rs76016088		21	21880100	0.027
	rs184686078		21	21880130	0.001
	rs138895664		21	21880157	0.073
	rs6517970	SNP 2	21	21880158	0.444
	rs202132081		21	21880159	0.064
	rs8131148	SNP 3	21	21880191	0.320
	rs6517971	SNP 4	21	21880231	0.420
	rs111754000		21	21880269	n/a

Additional documented variation in Microhap048 and MicroTetrad315. The “Clustered Allele Frequency” is the average in the 1000 Genomes data for the less frequent to vary rare allele at the SNP. The number of populations with data varies, and some have no frequency data available (n/a).

In the course of assembling a large number of microhap loci, we have come to recognize that a general nomenclature is advisable. Use of the specific SNPs involved is too cumbersome for use in ordinary text, and cross-referencing among publications from different laboratories will become difficult. Table 3 illustrates the heterogeneous nomenclature within just our lab because of different database searches and different priorities at different times. With reference databases that are searchable, we believe that a simple nomenclature with full database descriptions of the composition of the microhap and the population haplotype frequencies is possible. We hope to be able to implement such a nomenclature in ALFRED and FROG-kb as soon as there is some preliminary agreement among researchers interested in microhaplotypes. In the meantime, we are using our “LabNames” as provisional locus names that can be used to search ALFRED.

## Conclusions

While forensic researchers have demonstrated the utility of DNA analysis even in high-volume crimes [25], costs of sample collection and genotyping have engendered significant financial burdens. As new technologies of high-throughput DNA sequencing have been developed, the costs of obtaining large amounts of genetic data even from minute samples and/or degraded DNA have exponentially dropped. However, moving from a fragment-length-based analysis (such as the capillary electrophoresis systems commonly used to type CODIS markers) to a sequence-based analysis has been resisted. One common complaint from forensic scientists has been the problem of identifying DNA mixtures using di-allelic SNPs, even with sequence data; such mixtures can often be identified and resolved using STRP markers [26]. Detecting mixtures of DNA from two or more individuals in a forensic sample using di-allelic SNPs is a serious problem because no qualitative information is possible. While probabilistic estimates are possible using relative “intensities” of the alleles, that is less satisfactory. The problem disappears when SNPs are combined into a microhap locus with many alleles that can be unambiguously distinguished in a single sequence run. These multiallelic loci can have great stability, even surpassing STRPs, and are as easy to type by sequencing as individual SNPs. The relative value of a microhap for detecting mixtures qualitatively can be estimated using the effective number of alleles, symbolized as A_e_. Ranking loci by global average A_e_ values for loci we have already studied shows that a 4-SNP microhap can have very high values, but still less than five effective alleles. Even at an average A_e_ of 4, the probability of qualitative detection of a mixture is greater than 99% with just five such loci tested.

## Additional file

Additional file 1:
**Criteria used in our search of the 1000 Genomes data.** The dataset used for primary analysis for potential microhaplotypes is the Omni25_genotypes_2141_samples.b37 from 1000 Genomes Project.

## Abbreviations

SNP: single nucleotide polymorphism
LD: linkage disequilibrium
MPS: massively parallel sequencing
STRP: short tandem repeat polymorphisms
DIP: deletion/insertion polymorphisms
Indel: insertion-deletion polymorphisms
CODIS: combined DNA index system
A_e_: effective number of alleles
Microhap: microhaplotype
IBD: identity by descent
RFU: relative fluorescence units
ALFRED: allele frequency database <alfred.med.yale.edu>
FROG-kb: forensic resource/reference on genetics knowledge base <frog.med.yale.edu>
