# Supplementary material for: Criteria for selecting microhaplotypes: mixture detection and deconvolution
Source: Investig Genet. 2015 Jan 28;6:1. doi: 10.1186/s13323-014-0018-3 (PMC4351693; doi:10.1186/s13323-014-0018-3)
Supplement: Additional file 1: — Criteria used in our search of the 1000 Genomes data. The dataset used for primary analysis for potential microhaplotypes is the Omni25_genotypes_2141_samples.b37 from 1000 Genomes Project. [file 13323_2014_18_MOESM1_ESM.doc]

Additional file

Additional file 1 **Criteria used in our search of the 1000 Genomes data.**

The dataset used for primary analysis for potential microhaplotypes is the Omni25_genotypes_2141_samples.b37 from 1000 Genomes Project. Data were managed using plink and vcftools. We first removed SNPs with an allele with a global average frequency less than 0.05 (2,448,222 SNPs winnowed to 1,195,105 SNPs). We then selected all SNPs that have a neighboring SNP less than 200 bp away (1,195,105
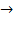
 295,702). We then removed duplicate SNP data (→292,419). Using a sliding window, we identified microhaplotype tetrads, where all four SNPs lie within 200 bp (→3,728 SNPs = 932 tetrads). We calculated for each SNP its heterozygosity for each population and an average global heterozygosity. We then averaged these global heterozygosities for an average-average heterozygosity for each tetrad. This value was used to rank order the tetrads. We also calculated the correlation between the frequencies for each SNP pair in the tetrad across populations; while imperfect, it was an easy estimator to minimize those SNPs that are in complete linkage disequilibrium, to remove those SNP pairs which behave as one locus. We averaged those correlations to generate an average correlation for each tetrad. After looking at the distribution of these heterozygosity and correlation metrics, we decided to remove those tetrads which had average-average heterozygosity <0.25 and abs (avg correl) >0.5. Our final working set of tetrads = 1,364 SNPs/341 tetrads.
